# The variant Creutzfeldt-Jakob Disease: Risk, uncertainty or safety in the use of blood and blood derivatives?

**DOI:** 10.1186/1755-7682-1-9

**Published:** 2008-06-23

**Authors:** Antonio Liras

**Affiliations:** 1 Department of Physiology, Biology School, Universidad Complutense of Madrid, Spain

## Abstract

It has been long since French physician Jean-Baptiste Denys carried out the first successful blood transfusion to a human being. Using bird feathers as canules, sheep blood was transfused to a young man. The patient died soon after Denys' treatment and Denys was accused of murder. In the XXI century, known as the biotechnology century, we face new challenges in Medicine. New emerging and reemerging diseases, such as Creutzfeldt-Jakob disease (CJD) or "mad cow disease" and its human variant (vCJD), challenge the biosafety aspects of a widely extended and extremely useful technique, that is, the perfusion of blood, of its derived components and of other pharmacological products obtained from plasma. To face these new challenges we need innovative prevention strategies.

## 

For over 20 years, hemotherapy has aimed at achieving the greatest safety in whole blood transfusions, blood components, or plasma derivatives, because availability of safe and reliable blood represents a crucial element for medical progress and for public health prevention. Concern arose at a time when approximately 1 out of every 100 blood units transmitted the human immunodeficiency virus (HIV) or hepatitis C virus (HCV).

Since Denys's first transfusion [1] there have been improvements in donor election and technological innovation in terms of both donor selection and detection of infectious agents using nucleic acid amplification procedures. Nonetheless, attention should be held to prevent future potential iatrogenic catastrophes, as it has been estimated, for instance, that 1 out of every 2000 platelet transfusions may contain infectious agents. Malaria and dengue are becoming new public health risks that also affect transfusion safety. Not so long ago, such infections were associated to exotic and sporadic travels, so that they were contained by keeping affected potential blood donors under quarantine for several months.

The current situation is very different. There is an exponentially increasing number of immigrants and emigrants who travel worldwide in a few hours, and *Trypanosoma cruzi *can arrive in 50% of immigrants coming from endemic areas. Moreover, in addition to *Trypanosoma*, *Babesia*, *Leishmania*, *Nile virus*, *avian flu virus*, *acute respiratory syndrome*, or the *Chikungunya virus *may also be rapidly transmitted. These emergent and re-emergent diseases extremely jeopardize transfusion safety [2].

The reality is that, as stated by Zessin [3], we are facing a livestock revolution and globalization, characterized by an increasing animal and foodstuff free trade. Modern life conditions are the result of globalization, which in many cases determines the prevalence of certain factors responsible for the so-called emergent diseases. Such factors may include certain ecological changes, such as those due to agriculture or economical development or resulting from climatic abnormalities, human demography or behavioral changes; also travel, trade, technology and industry, adaptation and changes in microorganisms, as well as a general lack of public health measures. With regard to pathogenic agents, a striking feature of emergent and re-emergent diseases is the current increase in the diversity of causative organisms, ranging from bacteria, rickettsias, fungi, protozoa, and helminths to proteins (prions), and including viruses.

Therefore, an epidemiological approach does not allow for long-term prediction and prevention with regard to the majority of new pathogens, but only provides a theoretical and coarse estimate of the risks for a given pathogen population. A minimum correlation between animal production, diseases of an animal origin, and human diseases requires reconsideration of concepts, methods, and structures. This allows for the implementation of new coordination measures in emergency situations which are prompted by diseases crossing species barriers.

## Transmissible spongiform encephalopathies

Bovine spongiform encephalopathy (BSE) has attracted attention to a group of diseases affecting animals and humans. Over time, these diseases have been given different names, some of which, alluding to the still insufficiently known purported causative agent, were scarcely rigorous from an academic viewpoint. Thus, reports spoke of diseases caused by slow viruses, viroids, virions, or non-conventional viruses and, more recently, of diseases caused by prions [4,5].

Thirty years ago, the most widely known condition, Creutzfeldt-Jakob disease (CJD), was a rare form of dementia unknown to many physicians. The name is now familiar because in 1996 bovine spongiform encephalopathy (BSE), widely known as "mad cow" disease, appeared. The potential association of the new human variant of Creutzfeldt-Jakob disease (vCJD) to BSE attracted the attention of health authorities, healthcare professionals, and the general population to this rare disease. Moreover, the possibility that there maybe a risk of transmission of vCJD through transfusions of blood and its derivatives has caused some social concern.

Transmissible spongiform encephalopathies (TSEs) are caused by infections agents, but may also occur as genetic or sporadic diseases. They are so called due to their main characteristics: spongiosis of the brain and transmissibility. Humans may experience Creutzfeldt-Jakob disease (CJD) in its sporadic, iatrogenic, and familiar forms; kuru; Gerstmann-Sträussler-Scheinke (GSS) encephalopathy; fatal familial insomnia (sporadic or familial) (FFI/SFI), and variant Creutzfeldt-Jakob disease (vCJD), which only affects humans.

## Creutzfeldt-Jakob disease

Creutzfeldt-Jakob disease (CJD) affects the brain, inducing its total neuronal destructuration, sponge-like appearance and complete loss of nerve function [6]. CJD is a degenerative disease with a fatal prognosis and an overall prevalence of approximately one in one million habitants. CJD may be sporadic (idiopathic), hereditary, or acquired. Sporadic CJD accounts for 80%–90% of cases worldwide. CJD is a transmissible neurological condition occurring mainly as the result of dura mater or corneal implants or treatments with growth hormone of a pituitary origin. The causative agent is a protein called "prion" (PrP). While hereditary and infectious cases have been documented accurately, in most reported cases the reason for prion occurrence is unknown. Typically, this leads to neurological symptoms affecting movement, as well as extreme drowsiness and clumsiness. In its severest grades, the disease may be associated to psychiatric symptoms, including a severe dementia state.

While in recent years much research has been done on the biochemical and replication aspects of prions, there is still much to be learned about the molecular mechanisms leading to nervous system neurodegeneration. However, it is highly possible that the cell biogenesis mechanisms of these proteins that control intracellular "traffic", folding, or extracellular targeting, are impaired [7]. The recent hypothesis that prion infection may require close contact between a given tissue, such as muscle, and infected neurons or nerve endings is also being tested [8]. Equally, it is not known whether a small amount of prion may be determinant to suffer the disease, though one may intuitively think that, as for any infection, occurrence of this disease may be dose-dependent or depend on other co-factors [9].

Three types of CJD have been reported to date: (i) sporadic or spontaneous, the most common type, accounting for 85% of all cases; (ii) familial or hereditary, involving a genetic component. A point mutation in the gene encoding for prior protein causes a defective folding of the protein, which precipitates and destructures the brain; and (iii) iatrogenic, when prions are acquired through the ingestion of meat or meat derivatives from affected animals, from transplants, or through the administration of blood or blood derivatives containing prions from humans with the disease or without any symptoms.

Variant Creutzfeldt-Jakob disease (vCJD) is the human form of "mad cow disease". Its causative agents are infectious proteins called prions, which are not inactivated by standard antiviral methods [10]. These new infectious agents are transmitted through the blood stream since they are adhered to the outer membrane of lymphocytes. In addition, due to their very small size, prions are barely retained by conventional sterilization filters. Moreover, they are proteins physiologically, rather than pathologically, present in the body with their normal structure. For this reason, they are not recognized as foreign by the body because their antigenic regions remain unchanged. Thus, an effective immunological rejection response is not mounted. The window or symptom-free period of this disease may be very long, up to 40 years, as shown by the fact that some children who received growth hormones extracted from pituitary glands of cadavers in the 60s are now developing vCJD. Consequently, due to the current unavailability of an *in vitro *test for detecting prions in blood, it is difficult to manage problems in terms of donation quality. Diagnosis may only be established on nerve tissue biopsies or post mortem biopsies.

Following the above, a blood donor may be in the asymptomatic period of the disease and be unaware of its presence. From the viewpoint of public health, such possibility, combined with the unavailability of screening tests for donors, turns these volunteers into high risk donors. Moreover, no curative treatment currently exists for the disease, so that a fatal outcome is to be expected. A prolongation of the disease's incubation time when prion accumulation in the brain is reduced by administering pentosan polysulphate by the intraventricular route has only been reported [11].

The normal form of the prion (PrP^c^), whose function is unknown, is located in the brain and other body regions in humans and many animals. The abnormal prion protein, or PrP^sc^, is chemically identical to the normal form, but has a different spatial conformation that makes it resistant to normal degradation processes in the cell. Furthermore, it induces its accumulation in several tissues, particularly in the central nervous system, where the most severe damage occurs [12]. Prions are also transmitted through the blood stream [13]. Disease progress is characterized by a neuronal tissue loss that causes a typical sponge-like appearance of the brain. A significant fact is the lack of a perceptible response of the immune system against the abnormal prion, despite its different spatial conformation. On the other hand, the abnormal prion protein is resistant to most methods used to inactivate bacteria and viruses. As a consequence, prions are not totally inactivated by heat, ultraviolet light, or other standard sterilization procedures, such as sodium hypochlorite at the usual concentrations. Simple autoclaving (a single cycle) is not sufficient to denature the abnormal prion protein present in surgical instruments.

The initial abnormal prion protein may occur spontaneously (a possible explanation for sporadic CJD), be linked to an inherited genetic abnormality in the PrP gene (familial CJD), or be acquired through biological material from infected people or plasma derivatives (iatrogenic CJD). It should be noted that a major proportion of patients with sporadic CJD and vCJD have the codon 129 polymorphism (methionine/methionine), a conditioning genetic factor [14]. This genotype, occurring in 40% of the general population, probably predisposes the conversion of normal prions into abnormal prions which are associated to the disease.

In cases where the disease is developed, a conformation change occurs within prions, so that prion proteins accumulate in neuron cells. This alters neuronal function, and ultimately leads to brain tissue vacuolization.

## Variant Creutzfeldt-Jakob disease (vCJD)

The first case of BSE in humans was diagnosed in 1994, ten years after the disease appeared in cows. In 1996, a new disease related to BSE, defined as a variant of CJD (vCJD), was first reported. Based on spatial and temporal coincidence, this condition was considered to possibly result from beef consumption, contrary to the idea that animal BSEs did not affect humans. In recent years, multiple experimental evidences have shown vCJD to be the manifestation of BSE in humans through what is already known as "a jump across interspecies barriers".

In 1992, the number of BSE cases peaked (to approximately 200,000 cows), and decreased to a minimum in 1998, leading to an epidemiological cascade of vCJD in humans due to the consumption of contaminated beef products. This was not limited to the United Kingdom, as cases have emerged in other countries due to the imports of sick animals or dietary supplements such as meat flours.

vCJD appears to be a disease with unique clinical and pathological characteristics, clearly different from those of classical or sporadic CJD. vCJD occurs in younger adults and develops more slowly over 14 months, as compared to the 4 months for classical CJD. Classical CJD occurs worldwide with an incidence rate of 1 case per million habitants yearly, while virtually all cases of vCJD have occurred to date in the United Kingdom. Classical CJD causes a rapid progression onto dementia or ataxia, while vCJD occurs as a psychiatric disease, with or without sensitive symptoms, that slowly evolves to the severe neurological stage. The final stages of both diseases are very similar [15].

Since the first case of vCJD, the following cases have been reported: 163 cases in the United Kingdom, 23 in France, 4 in Ireland, 3 in the United States, 3 in Spain, 2 in the Netherlands, 2 in Portugal, 1 in Italy, 1 in Canada, 1 in Saudi Arabia and 1 in Japan [16].

## Coagulation plasma factors and variant Creutzfeldt-Jakob disease in the treatment of hemophilia

In the 70s, hemophilia started to be treated with factors VIII or IX concentrates from human plasma fractionation. Subsequently, around the 90s, the first recombinant factors by genetic engineering and molecular biology were marketed. These were obtained from certain mammalian cells, conveniently modified with the corresponding factor VIII or factor IX genes.

From a pharmacological safety viewpoint, there are clear differences in the history of plasma and recombinant factors. Thus, only 11 years after plasma factors were firstly used, these had already triggered the great iatrogenic pandemic by the human immunodeficiency virus (HIV). A little later, in the 90s, the plasma factors caused infection by the then mistakenly called non-A, non-B hepatitis virus, which is currently known as hepatitis C virus (HCV). Both infections have caused thousands of deaths in hemophiliacs, decimating by more than a half the worldwide population of hemophiliac patients. Though these two types of viruses are inactivated in plasma concentrates, it is expected that they will cause many more deaths in the short and long term. This is due to the resistance to antiretroviral drugs and their cumulative adverse effects (mainly renal or pancreatic failure, or myocardial infarction because of increased cholesterol levels in relatively young patients), and because of liver cirrhosis development and hepatocarcinoma in 60% of patients not responding to current treatments for HCV.

In contrast to this tragic record of plasma products, recombinant factors offered hope and assurance for treating hemophilia but, above all, a safety that has been attested by the absence of any mild or severe adverse event, either infectious or of any other type, in the 18 years of use of these products for the treatment of hemophilia.

Since the final 80s, it was thought that the history of plasma factors could be changed by inactivating viruses with heat, and subsequently by dual inactivation methods at high temperatures as well as with solvents-detergents. However, this did not succeed. New threats have arisen and will arise with the so-called emergent diseases, prompted by the extraordinary demographic migration of humans, who can even cross interspecies barriers from animals to humans. This has caused plasma factors, which were and are very safe against viruses with a lipid envelope such as HIV and HCV, to become once more vulnerable to new infectious agents such as Creutzfeldt-Jakob disease.

Between 1997 and 2000, the United Kingdom National Blood Service and Health Protection Agency [17] reported the existence of blood donors who had developed vCJD after blood donation.

Since the initial emergence of vCJD in the United Kingdom, there was speculation about the theoretical risk of its transmission through blood and blood derivatives. While epidemiological evidence did not suggest that the sporadic form could be transmitted through blood, it was recognized that the new CJD variant could be potentially transmitted by such route because of its characteristics, as compared to the classical form, particularly with regard to involvement of the lymphoreticular system. This hypothesis took ground later, in December 2003, when contagion via a transfusion of packed red cells was confirmed in a patient. A second case sufaced in July 2004, followed by a third case in 2006 and by the most recent case in January 2007.

Concern arose in other countries, such as Spain, where the first case of vCJD in a female patient was reported in 2005. This case would have passed unnoticed had it not been for the fact that the woman, who died from the disease, had been a blood donor for several years. Her blood was used for plasma fractionation and for obtaining immunoglobulins, platelets and red blood cell concentrates, as well as concentrates of plasma factor VIII to treat hemophilia A. The Spanish pharmaceutical company that prepared this factor did not raise an alarm or, if it did, ensured that there was no risk of vCJD. All factor VIII batches prepared from plasma contaminated by prions were administered in their entirety to 300 hemophiliac patients.

## Risk of infection and development of the variant Creutzfeldt-Jakob disease through blood and plasma derivatives

Using animal models, it has been experimentally shown that prions are present in blood and are transmitted by this route [12,13]. In addition, white blood cells —which may be found in packed red cells and other blood components for transfusion use— have aninfectivity similar to plasma [18]. Therefore, there is a risk through surgical instruments, transplants, blood transfusions, or treatment with products derived from contaminated plasma.

According to Farrugia [19], the risk parameters include essentially: (i) number of donations; (ii) proportion of blood donors infected by vCJD;(iii) plasma volume per donation; (iv) number of infective vCJD units per mL of plasma; (v) number of product units prepared in the production process; (vi) logarithmic reduction in the number of infective units during the production process; and (vi) amount of product used by the patient. To summarize, the potential low infection risk of such concentrates is partially due to two factors, namely the dilution of a contaminated donation with thousands of "clean" donations in the same plasma batch, and the manufacturing process itself, provided this includes steps that remove the infectious agent such as leukodepletion, selective precipitation, nanofiltration, and ion exchange chromatography.

No data quantifying the risk of infection through plasma products may be provided due to the lack of a wealth of information about pathogenic agents that cause the disease. However, risk estimations may be drawn based on highly complex theoretical epidemiological models. Therefore, such estimations will only serve as a guide for taking the necessary public health precautions for preventing any potential transmission of vCJD, or for implementing the clinical and rational monitoring of patients who have received prion-contaminated products.

It is believed that when plasma derived products from donors who subsequently developed vCJD has been received, the risks are high. In the autumn of 2003, the consulting company *Det Norske Veritas *(DNV) performed a risk estimation of prion infection when plasma factors are used [20]. The Spongiform Encephalopathy Advisory Committee (SEAC), the Committee on the Microbiological Safety of Blood and Tissues, and the Committee on Safety of Medicines accepted the risk data, and currently it continues to be regarded as the only reference data [19].

Despite effective inactivation methods used for plasma factors, it cannot be currently stated that these products are totally safe, as they are always prepared from thousands of blood donors and emergent diseases cannot be controlled under these circumstances. Today, plasma factors are only safe against viruses having a lipid envelope, because these viruses are sensitive to procedures using solvent-detergent and heat. This is not the case for viruses not having a lipid envelope, such as hepatitis A virus (HAV) or parvovirus B19, or other emergent similar viruses which may emerge. Indeed, current viral inactivation procedures are not effective for prions that are resistant to all these methods. By contrast, such circumstances do not take place with factors of recombinant origin.

While risk estimation varies depending on prion clearance steps during plasma fractionation and on the different authors and the different methods used to prepare blood derivatives [21-24], factor VIII and factor IX concentrates obtained from cryoprecipitates and cryosupernatants respectively are considered to involve a middle to high risk for vCJD if plasma was contaminated by prions. It is understood that the albumin used as stabilizer for recombinant factor VIII concentrates involves a low risk.

The risk is low if clearance or risk reduction steps used during the concentrate preparation process and which attenuate prion levels are considered. It is known that the time or symptom-free period before the first symptoms of the disease occur may be very long, up to 10 or 20 years, and also that vCJD is actually transmitted through blood, as shown in animal models. Alternative measures consisting of treatments not involving use of plasma-derived products, such as recombinant products, must therefore be used, at least until the intimate mechanisms —prion levels, transmission routes— are known and the reliability of prion clearance procedures is established. A test for detecting prions in blood in order to rule out blood donors who are vCJD carriers would be determinant. What is clear, and this is no novelty in medicine, is that risks exist, and in no case could they be assumed to be non-existent.

However, it should be noted that no case of vCJD has been reported to date in hemophiliacs who have received concentrates from plasma containing prions. Nonetheless, in medicine the absence of evidence does not represent an evidence of absence [25]. Equally, the possibility that some cases have been overlooked because of the current inadequate understanding of the disease and the unavailability of an adequate diagnostic method should not be dismissed.

It is still very difficult to conclude on the possibility of developing vCJD following a prion infection because there are no adequate statistical data to risk predictions and no molecular bases are available to establish etiopathogenetic mechanisms.

## Prevention measures

Using data collected from a variety of animal species in experimental models, the World Health Organization prepared prevention measures from the classification of the grades of infectivity of organs, tissues, and fluids.

In principle, general measures for any type of infection must be taken, such as advising the general public against the consumption of meat or products derived from infected animals. In any good clinical practice, measures appropriate directed to "biosafety" should also be taken, including the decontamination of surgical materials in general and of dentistry materials in particular. Whenever possible, disposable materials should be used and in spite of their higher costs innovative prion inactivation procedures should be implemented [26-29].

Based on current knowledge about spongiform encephalopathies, there is no evidence of a transmission risk from regular close contact through the skin, saliva, or intercourse. Nonetheless, for public health reasons it is advisable to avoid close contacts or ovule or sperm donation in prion infected patients.

Despite the above and from the epidemiological viewpoint of an infectious disease, the most appropriate action is to implement or develop strict pharmacovigilance measures, in this case hemovigilance measures [30,31], that will include the detection, classification, and analysis of the adverse effects of transfusions of blood or its derivatives. In this way, their causes may be corrected and their recurrence may be prevented. In this respect, the European Parliament Directive 2002/98/EC [32] included in its chapter V the obligation of all Member States to take any actions required to guarantee the traceability from donor to receptor and vice versa, as well as to report any serious adverse effects and reactions related to the drawing, verification, treatment, storage, and distribution of blood and blood components which may influence their quality and safety. Any serious adverse reactions that may occur during or after a transfusion and that may be attributed to the quality and safety of blood and its components should also be reported.

In order to achieve the above objectives, it is essential to follow-up both blood donors, potential asymptomatic vCJD patients, and patients who have received blood contaminated by prions. This could be either as whole blood, plasma, or derivatives, and blood components such as packed red cells or platelets, but mainly concentrates of coagulation factors, as these products have a very high concentration of proteins that may include prions.

As regards blood, the requirements of traceability and reporting of serious adverse reactions and effects of blood and blood components must be considered, together with reliable clinical practice and optimum donor selection, leukodepletion —or white blood cell removal from blood—, registration of blood donors, and information to receptors of blood or its derivatives [33-35]. People who have lived for more than one cumulative year in the United Kingdom —England, Scotland, Wales, Northern Ireland, Isle of Man, and Channel Islands— during the period from 1980 to 1996, both inclusive, can be excluded as risk donors. Universal blood leukodepletion is required [36] because it has been clearly shown that prion levels are very high in the lymphoreticular system [37], and the use of other procedures to remove prions from blood derivatives, such as several heating cycles, nanofiltration, and ion exchange chromatography should be urged [38].

## Current and future alternatives for maximum prevention

There are three main axioms regarding blood: first, blood will always be a scarce good that will have to be donated; secondly, there is no zero risk for a blood receptor; and thirdly, since blood is a biological product, it will never be totally free from contamination by currently known viruses, bacteria, or prions or by other still unknown agents. In such circumstances, and since blood will continue to be required, an immediate objective is to try and administer the so-called "red blood" at hospitals in the safest and most adequate way [39]. It should also be noted that while emergent diseases represent a real and very current concern, blood donation is an inevitable fact because of the low offer of donations and the great demand for blood and its derivatives [40].

These reasons, simple and complex at once, suggest a number of actions to ensure the safety of blood and its derivatives. First, this involves the implementation of measures aimed at donor selection based on epidemiological and demographic factors related to given diseases and on serologic tests negative for different infections possibly detectable in blood. Secondly, drugs that are not derived from plasma should be used, such as recombinant products, which have not caused side effects after almost 25 years of use and therefore have become a highly effective and safe therapeutic alternative [41]. In this sense, many countries are implementing health policies aimed at recommending and encouraging the use of recombinant medicines, provided that they are not contraindicated and that such use is permitted by social and economic factors.

On the other hand, international consensus has been reached about the different alternatives to allogeneic blood transfusion [42]. Actual examples of such alternatives include the administration of *activated recombinant factor VII*, particularly useful for decreasing bleeding and/or transfusion requirements in various medical or surgical procedures where uncontrolled massive bleeding occurs with conventional methods. Another example is *aprotinin*, a dose-dependent antifibrinolytic that inhibits trypsin, plasmin, and plasma and tissue kallikrein. Furthermore, *desmopressin *increases platelet adhesion —by increasing expression of the platelet GPIb receptor— and plasma levels of factor VIII and von Willebrand factor from its production sites in the endothelial cells of hepatic sinusoid. Equally, recombinant *erythropoietin *stimulates erythropoiesis by inhibiting apoptosis of erythroid precursors and promotes proliferation and maturation into erythrocytes. Finally, *perfluorocarbons *is useful for intravascular volume replacement, and to fix gases such as O_2_; and *perioperative recovery of autologous blood *to be returned to the patient as packed red cells in saline.

In addition, pharmaceutical companies are fighting a battle against the clock to find a blood substitute —"artificial blood"— using biotechnological procedures. The so-called "masked" red blood cells and fluorocarbon compounds are examples of future substitutes for red blood cells carrying oxygen into tissue. In turn, fragments of platelets conjugated with serum albumin represent a future possibility for dispensing with platelet infusion in cases of thrombocytopenia [43]. More recently, polyhemoglobins conjugated to antioxidant enzymes, coated by lipid membranes, have opened up a new perspective for the future [44].

## Final conclusion

The history of medicine has undoubtedly shown that a zero risk does not exist in any medical practice. Therefore, the greatest possible number of public health precautions should be taken even if statistics suggest that there are minimum risks, as it happened in 1979 for AIDS and may occur today for vCJD. *A posteriori*, those predictions have been shown to be clearly wrong, and HIV has infected approximately 65 million people, of whom more than 25 millions have died from AIDS [45].
